# Identification and control of booming noise of thin-walled metal plates of commercial vehicle body subjected to high-speed airflow

**DOI:** 10.1371/journal.pone.0319984

**Published:** 2025-03-31

**Authors:** Yuanshao Wang, Bao Zhang, Xiaoping Su, Liguo Zang

**Affiliations:** 1 School of Mechanical and Power Engineering, Nanjing Tech University, Nanjing, China; 2 Nanjing Tech University Pujiang Institute, Nanjing, China; 3 Naveco Automobile Co., Ltd, Nanjing, China; 4 Nanjing Institute of Technology, Nanjing, China; CINVESTAV IPN: Centro de Investigacion y de Estudios Avanzados del Instituto Politecnico Nacional, MEXICO

## Abstract

The thin walled metal plates (TWMP) on top of a commercial vehicle body are easy to produce vibration and booming noise under the action of high-speed airflow, which reduces the sound quality inside the vehicle. Therefore, the effective control of the noise of TWMP is very important for vehicle comfort performance. Based on experimental, finite element, dynamic and fluid techniques, a method for identification, analysis and optimization of TWMP booming noise under high-speed airflow is proposed in this paper. The problem frequency, the noise source and the cause of booming noise inside the vehicle are identified by amplitude-frequency characteristics analysis and modal analysis. By establishing the dynamic model of damping patch, the matching method of damping patch is proposed. The simulation results are in good agreement with the experimental results, which shows that the method is correct. And the damping coefficient, attachment position and attachment area of the damping plate can be analyzed quickly and accurately by using the method, and the vibration and noise of TWMP can be reduced obviously.

## Introduction

The structure of commercial vehicle body is mainly composed of the body frame and outer covering parts. The body frame acts as a support. The outer covering parts not only play the role of appearance decoration, but also are the force structures. The outer covering parts are made of multiple TWMPs welded together. The TWMP is mostly rectangular and its thickness is about 0.7 mm. Under external excitation, TWMP is prone to vibration, resulting in booming noise, which seriously affects the sound quality and comfort inside the vehicle [1,2]. Therefore, it has important engineering application value for the study of TWMP dynamic characteristics.

The booming noise is mainly caused by TWMP vibration. The excitation sources mainly include: road unevenness excitation, wheel rotation excitation and high-speed airflow excitation. The road unevenness excitation is transmitted to the body through “tire - suspension - the body rubber support “, which causes the body vibration. By improving the damping performance of suspension system, the vibration can be reduced effectively, and the problem of booming noise can be solved [3]. The wheel rotation excitation frequency coincides with the natural frequency of the body frame, which causes the body resonance and TWMP vibration. The problem of the booming noise can be solved effectively by reducing wheel dynamic unbalance and increasing the stiffness of body frame [4]. Under high-speed driving conditions, the high-speed airflow directly acts on the TWMP, causing the TWMP to produce forced vibration and make booming noise. In order to solve the problem caused by high-speed airflow, the common methods are to change the body shape or increase the stiffness of TWMP [5]. Because of the technical difficulty, long cycle and high cost, the body shape is difficult to change. The stiffness can be improved by increasing the thickness of the TWMP, adding rib plates or attaching reinforcing plates. Increasing the thickness or adding rib plates is not conducive to lightweight design of the body. Attaching reinforcing plates needs baking process, which is difficult for the long wheelbase body, and the reinforcing plates are easy to fall off.

In order to reduce the vibration and booming noise of TWMP, it is necessary to study the dynamic characteristics of TWMP. The booming noise is related to the stiffness, material, boundary condition and incentives[6,7]. For example, G. Petrone [8] analyzed the influence of thickness, damping and excitation mode of rectangular TWMP on the mechanical properties through numerical and experimental methods. Tran [9] studied the effects of fixed boundary conditions and simply supported boundary conditions on the vibration performance of TWMP. The results showed that the reasonable design of the structure is the basis of vibration and noise control. However, the effect of acoustic-structure coupling is not considered in references [8,9].

The vibration and noise analysis process of TWMP is “noise identify - contribution analysis - optimization”. In order to accurately analyze the vibration and booming noise of TWMP, it is necessary to conduct acoustic--structure coupling analysis, which can establish the relationship between vibration and noise, and is beneficial to the identification and contribution analysis of noise [10,11]. For example, Luo [12] studied the vibration-acoustic performance of rectangular TWMP under the acoustic-structure coupling condition, and the research results showed that the coupling had a significant effect on the modal results, which affected the dynamic characteristics of TWMP. Shu [13] proposed a new sensitivity analysis and optimization method based on the acoustic-structure coupling model, which effectively reduced the abnormal noise through material optimization.

The problem frequency of booming noise is identified according to the amplitude-frequency characteristics, but the noise source cannot be identified by this method. The contribution analysis of TWMP mainly uses numerical method or experimental method, and contribution analysis is performed based on vibration transfer function (VTF) and noise transfer function (NTF) [14,15]. The high-speed airflow directly acts on the surface of TWMP, so the VTF and NTF methods cannot be applied to this condition. In the optimization of TWMP, damping material [16] is mainly used to reduce noise. However, the selection of attachment position and attachment area of the damping plate is mainly based on engineering experience, and there is no relevant theory.

Based on the current research status, a method of identification, evaluation, analysis and optimization of TWMP booming noise under the action of high-speed airflow is proposed in this paper. And the main contents and innovations are as follows:

(1) The methods of frequency identification of booming noise and noise source identification under the action of high-speed airflow are presented.
(2) The booming noise inside the vehicle is analyzed and optimized by using experiment, finite element, dynamics and fluid techniques.
(3) The matching method of damping plate is proposed by establishing the dynamic model of damping plate.

## Test analysis

### Test description

**Fig 1:**
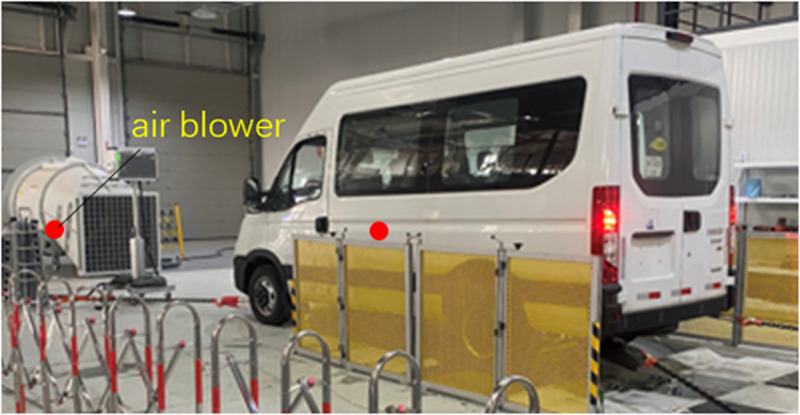
High-speed airflow field test.

When the vehicle is traveling on a flat road at a speed of more than 115 km/h, there is booming noise inside the vehicle. The higher the speed, the more serious the noise. And the noise comes from the top of the vehicle body and is affected by wind speed. The noise may be caused by the vibration of the body sheet metal caused by high-speed airflow. In order to comprehensively and accurately analyze the causes of the booming noise inside the vehicle, the following tests were carried out:

(1) Fixed working condition (gearbox in neutral) test: Firstly, the working condition test with uniform acceleration rev condition was carried out, the rev range is 800rpm-3600rpm, and there was no booming noise in the vehicle; Secondly, the uniform rev condition test was carried out, the rev range is 800rpm-3600rpm, and the test was carried out every 200rpm increase, and there was no booming noise in the vehicle. The results showed that the booming noise inside the vehicle is not caused by the engine.
(2) Chassis dynamometer test: The vehicle was placed on the chassis dynamometer, and the engine was turned off. The chassis dynamometer drove the rear wheel to drive the transmission shaft to rotate. Under the conditions of uniform speed and uniform acceleration, with the speed range was 40km/h-120km/h, there was no booming noise inside the vehicle. The results showed that the booming noise in the vehicle was not caused by the transmission shaft.
(3) Impact vibration test: The vehicle was placed on the vibration test bench, and the engine was turned off, the transmission shaft was not rotating. The road spectrum data (speed range from 40km/h to120km/h) was used to drive the test bench. When the corresponding speed was 100km/h, there was the booming noise in the vehicle, but the booming noise was not obvious. The results showed that the road excitation was not the main cause of the booming noise inside the vehicle.
(4) Environmental chamber test: The vehicle was placed in the environmental chamber, as shown in Fig 1, and the engine was turned off, the transmission shaft was not rotating. The air outlet of the blower was facing the front of the vehicle. Wind speed was set according to different speed, which was used to simulate the driving state of the vehicle. When the wind speed was 115 km/h, there was the booming noise in the vehicle, and the faster the wind speed, the more obvious the noise. The test results showed that the booming noise inside the vehicle was caused by the high-speed air flow.

### Noise identification

**Fig 2:**
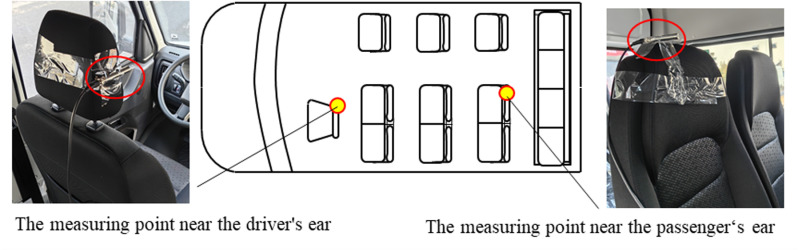
The noise test.

**Fig 3:**
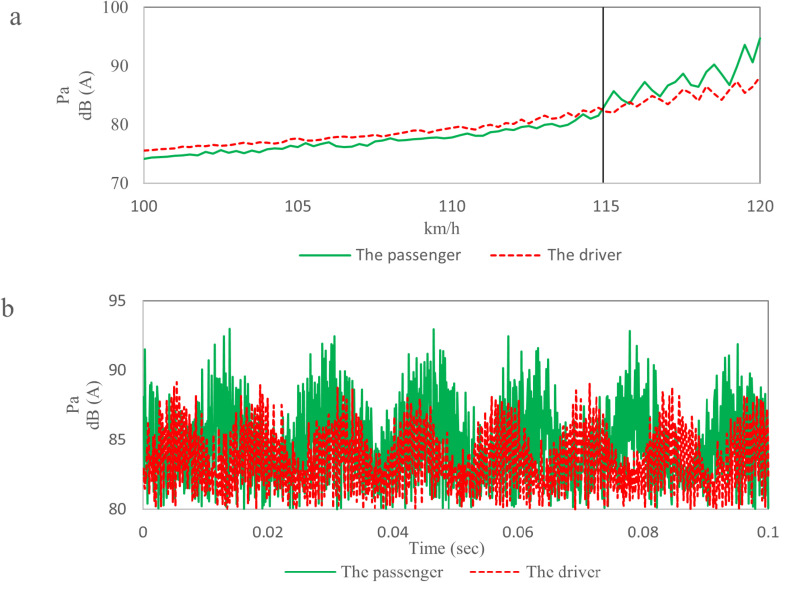
Noise data inside vehicle.

Noise sensors are arranged beside the driver’s ear and the passenger’s ear respectively, and LMS equipment is used to collect noise data, as shown in Fig 2. The wind speed is uniformly accelerated from 100 km/h to 120 km/h. The noise data under uniform acceleration condition is shown in Fig 3a. When the wind speed <  115 km/h, the noise beside the driver’s ear is greater than that beside the passenger’s ear, the average difference is 1.2 dB, and the noise data of the two positions vary gently. When the wind speed ≥ 115 km/h, the noise beside the passenger’s ear is greater than the noise beside the driver’s ear, the faster the wind speed, the greater the difference. And there are fluctuations in both noise test data, and the noise data beside the passenger’s ear fluctuates obviously. The faster the wind speed, the greater the fluctuation. When the wind speed is 115 km/h, a slight booming noise begin to appear inside the vehicle. When the wind speed is 120 km/h, the noise is very obvious, which has seriously affected the comfort performance.

The noise data at a wind speed of 120 km/h is shown in Fig 3b. The maximum noise beside the driver’s ear is 89 dB and the maximum noise beside the passenger’s ear is 93 dB. The noise data has “beat” phenomenon and has obvious periodicity, which shows that the body has resonance problems. The phase of the driver’s ear noise data lags behind that of the passenger’s ear noise data. The phenomenon indicates that the source of noise inside vehicle comes from the rear of the body. In order to identify the problem frequency of the noise, the autocorrelation analysis of the noise data is carried out [17].

Autocorrelation analysis describes the correlation degree of the data at different times, which is used to extract periodic signals in data and attenuate random interference signals. x(t) is a random vibration signal at time t, and x(t+τ) is the random vibration signal at time t+τ, the sampling length is *T*, and the autocorrelation function of the signal is


$$\begin{array}{c} R_{x}(\tau )=E\left[x(t)x(t+\tau )\right]\\ \text{=}\underset{T\to \infty }{\lim}\frac{1}{T}\int_{0}^{T}x(t)x(t+\tau )dt \end{array}$$ (1)

The autocorrelation function is expressed in discrete form as


$$R_{x}(k)=\frac{1}{N}\sum_{i=1}^{N-k}x(i)x(i+k)$$ (2)

where, τ stands for time difference, N stands for data length, and k stands for data interval.

The sinusoidal signals are x_0_(t), x_1_(t) and x_2_(t), the interference signal is ν(t), the corresponding energy of the four signals are E_0_, E_1_, E_2_ and E_3_, and there is E_0_ > E_1_ > E_2_ > E_3_. x(t) is the superposition signal of the four signals. During the autocorrelation analysis of vibration signals, there is no correlation between the interference signal and the periodic signal, and there is no correlation between the different frequency periodic signals. According to the conclusion, the autocorrelation function of input signal x(t) is


$$\begin{array}{c} R_{x}(\tau )=E\left[x(t)x(t+\tau )\right]\\ \text{=}\underset{T\to \infty }{\lim}\frac{1}{T}\int_{0}^{T}x(t)x(t+\tau )dt\\ \text{=}\underset{T\to \infty }{\lim}\frac{1}{T}\int_{0}^{T}\left[x_{0}(t)+x_{1}(t)+x_{2}(t)+\nu (t)\right]\cdot \\ \left[x_{0}(t+\tau )+x_{1}(t+\tau )+x_{2}(t+\tau )+\nu (t+\tau )\right]dt\\ =\underset{T\to \infty }{\lim}\frac{1}{T}\int_{0}^{T} [x_{0}(t)x_{0}(t+\tau )+x_{1}(t)x_{1}(t+\tau )+\\ \left.x_{2}(t)x_{2}(t+\tau )+\nu (t)\nu (t+\tau )\right]dt \end{array}$$ (3)

**Fig 4:**
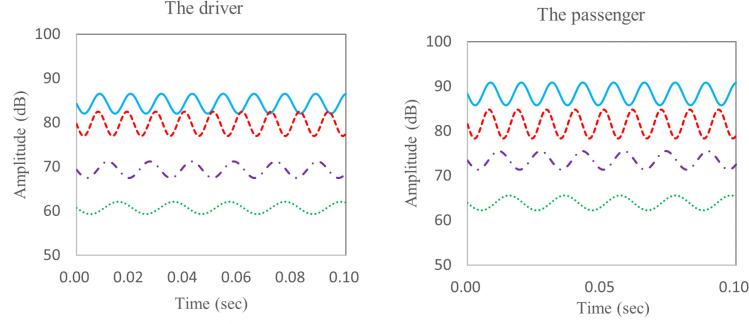
Results of autocorrelation analysis.

According to Eq. (3), the noise data in Fig 3b was analyzed by autocorrelation and the noise curves of four main frequencies were separated, the autocorrelation analysis results of the noise data in Fig 3b are shown in Fig 4. The blue line is the noise curve with a frequency of 87.6Hz, the red line is the noise curve with a frequency of 93.9Hz, the purple line is the noise curve with a frequency of 65.4Hz, and the green line is the noise curve with a frequency of 48.6Hz. The amplitude of noise corresponding to each frequency is shown in Table 1. Therefore, the main frequencies are 87.6Hz and 93.9Hz.

**Table 1:** Noise at the key frequencies.

Frequency (Hz)	48.6	65.4	87.6	93.9
Driver noise (dB)	62.1	71.2	86.5	82.5
Passenger noise (dB)	65.6	75.5	90.8	84.9

**Fig 5:**
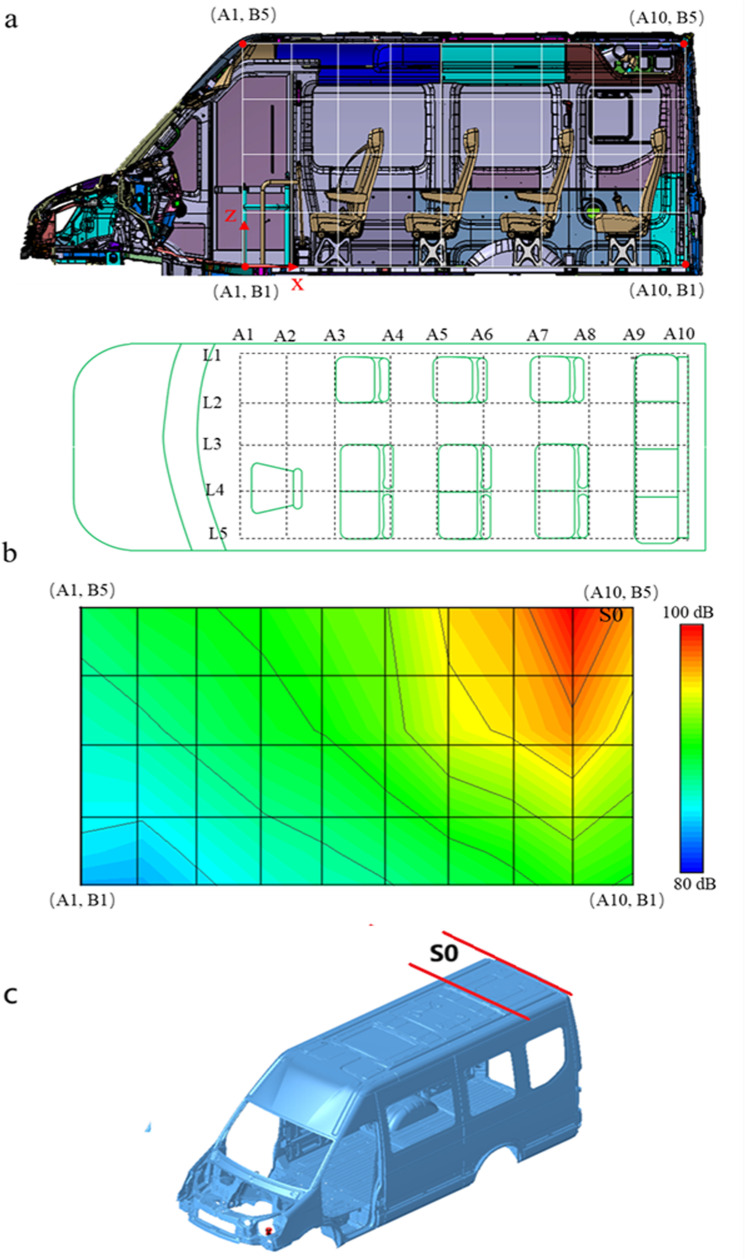
Sound field test (a) the model; (b) the test result of XOZ plane inside vehicle; (c) the S0 region.

In order to analyze the noise source, it is necessary to analyze the sound field inside the vehicle. The origin of the vehicle coordinate system is the center of the two front wheels, the X-axis points to the rear of the vehicle, the Y-axis points to the right, and the Z-axis points upward. In order to analyze the sound field distribution in the vehicle more comprehensively, when the test was carried out, the vehicle body was divided into 5 columns (L1-L5) in the Y directions, and the body was divided into 10 rows (A1-A10) in the X directions, and divided the body into 5 rows (B1-B5) in the Z direction, as shown in Fig 5a. In order to analyze the cause of the booming noise inside the vehicle, the noise test was carried out at 120km/h wind speed. The noise data of different area was measured, and the sound field inside the vehicle was reconstructed according to the test results. The results showed that the distribution law of sound field of L1-L5 was very similar, and the L3 sound field was the most obvious. The sound field distribution of L3 was shown in Fig 5b. In the X direction, the noise at the rear of the body is greater than that at the front. In the Z direction, the noise in the upper part of the body is greater than that in the lower part. The maximum noise appears in the region S0 (A8B5-A10B5) which is shown in Fig 5, and decreases around the region S0 as the center, the sound field shows an obvious stepped shape. The analysis results show that the noise source is in the region S0. During the test, the sealing condition of the body was good and the noise leakage was small. Under high-speed conditions, wind noise had an obvious impact on the noise inside the vehicle. The higher the speed, the greater the wind noise, but the airflow noise itself will not produce significant fluctuations. However, the noise data in Fig 3 showed a jumping phenomenon after 115km/h, resulting in booming noise. Therefore, the booming noise inside the vehicle was caused by the structural radiation noise.

The analysis results showed that the booming noise inside the vehicle is not caused by the sheet metal on the left and right sides of the body, but by the sheet metal on the top of the rear of the body.

**Fig 6:**
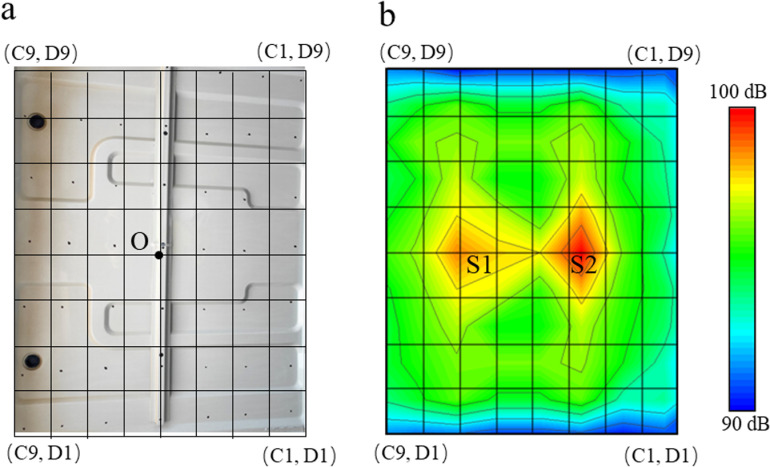
The sound field test of the TWMP in region S0.

The surface of the TWMP in region S0 is divided into 64 regions, as shown in Fig 6a. And Fig 6b shows the sound field of the TWMP. There are two noise sources on the surface of TWMP, namely, region S1 and region S2, and the noise in region S2 is greater than that in region S1. Region S1 and region S2 are distributed symmetrically along the Y-axis.

**Fig 7:**
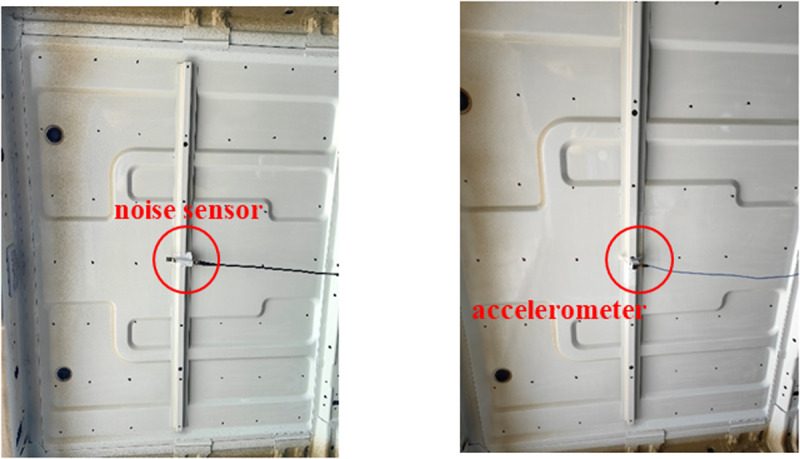
Vibration and noise test.

In order to identify the problem frequencies of booming noise, noise and vibration analysis of TWMP is necessary. A noise sensor and an acceleration sensor are installed on the TWMP surface at point O, as shown in Fig 7. LMS.Test Lab is used as the test equipment, the vibration acquisition frequency range is 0-100 Hz, and the sampling resolution is 0.1 Hz. The frequency range of noise acquisition is 0-100 Hz, and the sampling resolution is 0.1 Hz. The test wind speed is 120 km/h.

**Fig 8:**
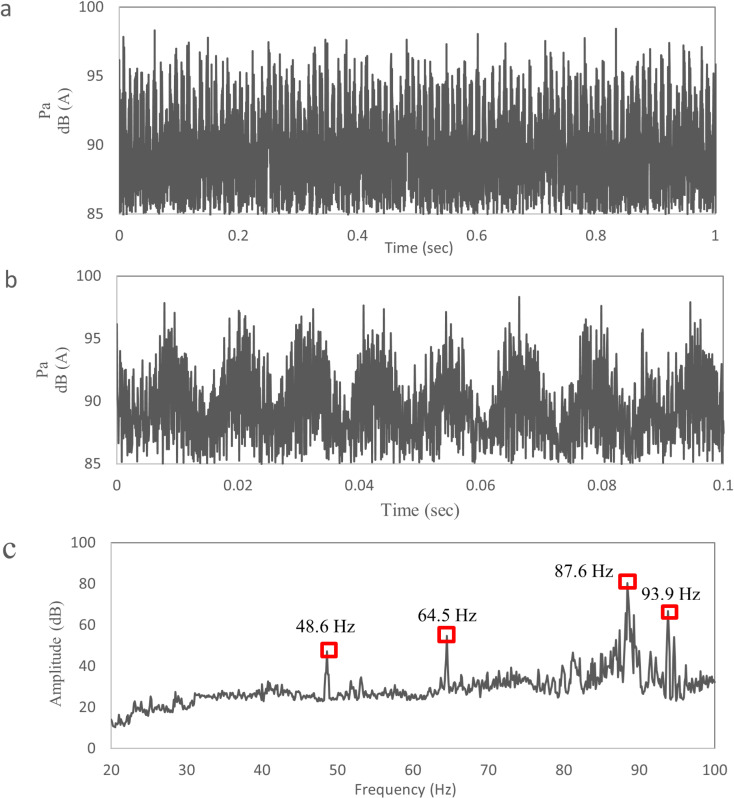
Booming noise data of TWMP.

Fig 8a shows the booming noise of TWMP in the region S0 at 120 km/h wind speed, and the noise ranges from 85.0 dB to 98.4 dB. Fig 8b shows the time domain data within 0.0-0.1 seconds. There is an obvious “beat” shape, which causes sound pressure fluctuation and reduce sound quality inside the vehicle. Fig 8c shows the frequency domain data, and the peak frequencies are 48.6 Hz, 64.5 Hz, 87.6Hz and 93.9 Hz. The primary frequency that has the greatest effect on the booming noise is 87.6Hz, and the corresponding amplitude is 80.5dB. The corresponding amplitudes of 48.6 Hz, 64.5 Hz and 93.9 Hz are 47.3 dB, 54.8 dB and 67.8 dB respectively.

**Fig 9:**
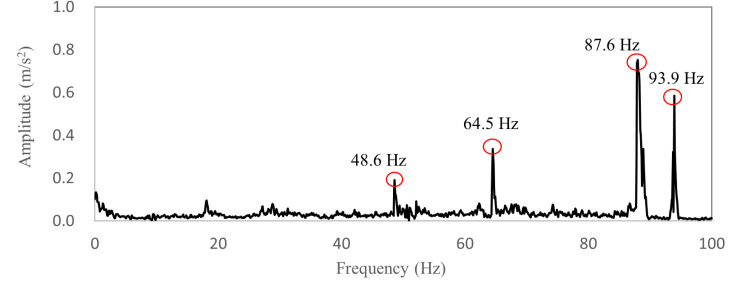
The vibration test data of TSWM.

Fig 9 shows the vibration amplitude-frequency curve of the TWMP. The peak frequencies are 48.6 Hz, 64.5 Hz, 87.6Hz and 93.9 Hz. The maximum amplitude is 0.754 m/s^2^ at 87.6 Hz, the second largest amplitude is 0.754 m/s^2^ at 93.9 Hz. Therefore, the key frequencies of TWMP vibration are 87.6Hz and 93.9Hz. The vibration frequencies match the noise frequencies, which indicates that the booming noise inside vehicle is caused by the vibration of TWMP under the action of high-speed airflow.

### Reason analysis

In order to prevent the booming noise, TWMP modal frequencies should be kept away from acoustic cavity modal frequencies. The requirements include: the top TWMP modal frequency should be different from Z direction modal frequency of the acoustic cavity, the side TWMP modal frequency should be different from Y direction modal frequency of the acoustic cavity, and the modal frequency of the front and back TWMP should be different from X direction modal frequency of the acoustic cavity.

**Fig 10:**
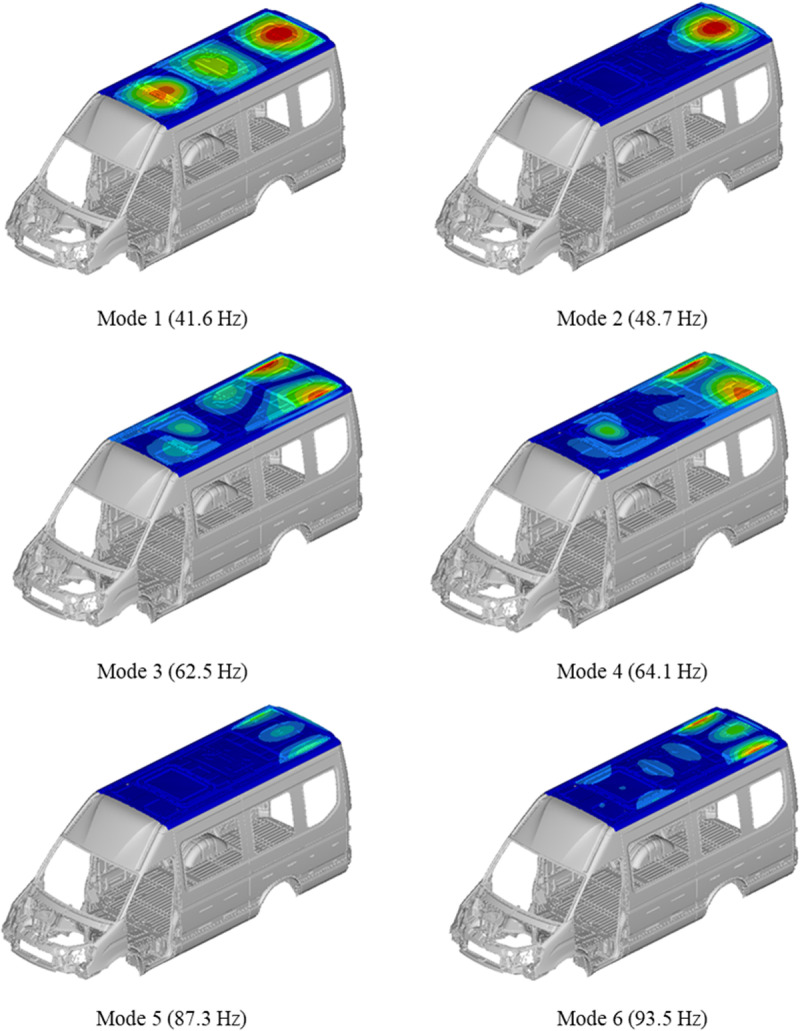
The modal of TWMP.

**Fig 11:**
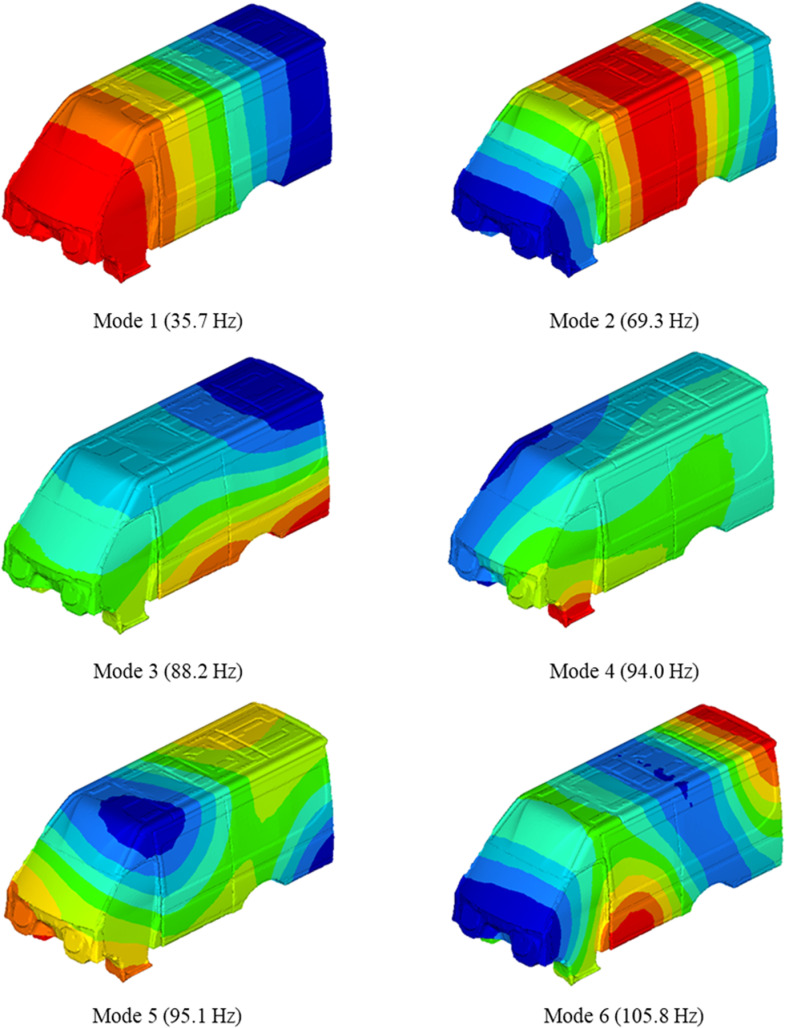
The modal of acoustic cavity.

In order to further analyze the causes of booming noise inside the vehicle, TWMP structural modal analysis [18,19] and acoustic cavity modal analysis [20] are carried out. In this part, the finite element method is used to create the body model and the acoustic cavity model. The structural mode simulation results are shown in Fig 10, and the acoustic cavity mode simulation results are shown in Fig 11.

The comparison between TWMP mode and acoustic mode shows that the fifth mode of TWMP coincides with the third mode of acoustic cavity, and the sixth mode of TWMP coincides with the fourth mode of acoustic cavity. The above results cause acoustic-structure coupling vibration, which produces booming noise inside the vehicle. The third mode of the acoustic cavity is the Z direction mode, so the influence of the fifth mode of the TWMP is the most important. The data in Table 1 and Fig 9 also indicates that the fifth modal of the TWMP has the most obvious influence on booming noise.

The vibration differential equation of a rectangular thin plate under the dynamic load q(x,y,t) is [21]


$$\frac{Eh^{3}}{12(1-\mu^{2})}(\frac{\partial^{2}}{\partial x^{2}}+\frac{\partial^{2}}{\partial y^{2}})\omega +m\frac{\partial^{2}\omega }{\partial t^{2}}=q(x,y,t)$$ (4)

where, E stands for elastic modulus, μ stands for poisson’s ratio, h stands for thickness of the plate, ω stands for the natural frequency of the plate, and m stands for the mass per unit area of the plate.

According to Eq. (4), the dynamic deflection of the plate is


$$\xi =\sum_{m=1}^{\infty }\sum_{n=1}^{\infty }\left[A_{mn}\cos\omega_{mn}t+B_{mn}\sin\omega_{mn}t+\tau_{mn}(t)\right]W_{mn}(x,y)$$ (5)

where, Wmn(x,y) stands for the mode function of the plate, *ω*mn stands for the natural frequency of the plate, Amn and Bmn stand for specific coefficients, and tmn (t) stands for any particular solution.

Eq. (5) shows that the vibration of the plate is a superposition of deformation at multiple modal frequencies. Therefore, the vibration of the TWMP analyzed in this paper is not only affected by the fifth mode and the sixth mode, but also by the first, second, third and fourth modes. When the wind speed is less than 115 km/h, there is no booming noise inside the vehicle, which indicates that the booming noise is not only related to the acoustic-structure coupling, but also related to the vibration intensity of the TWMP. Therefore, there are two ways to solve the problem of booming noise. Method 1 is to change the modal frequency of TWMP, and Method 2 is to attenuate the vibration amplitude of TWMP.

### Deformation analysis of TWMP

**Fig 12:**
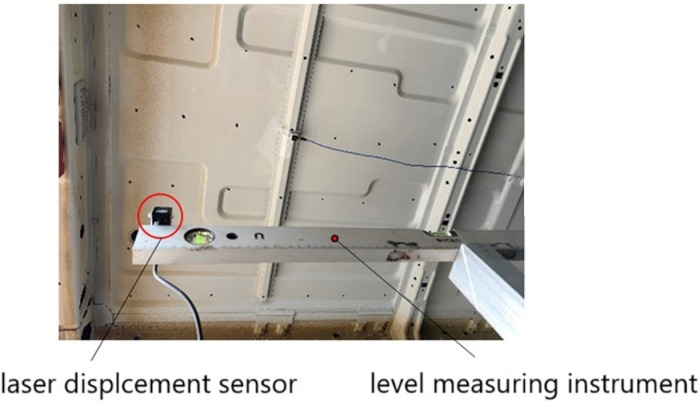
Deformation test of TWMP.

The modal frequency of TWMP can be changed by the method 1 (increasing the thickness or adding rib plates). However, the method 1 is not conducive to the lightweight of the body, but also increases the cost. Therefore, the method 2 is chosen to solve the problem of booming noise in the paper. The vibration intensity is expressed by the maximum deformation of TWMP. Fig 12 shows the deformation test of TWMP. The test equipments consist of laser displacement sensor and a level measuring instrument.

**Fig 13:**
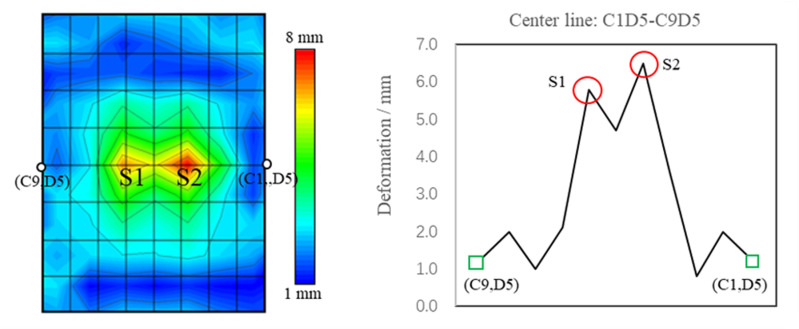
The deformation data of TWMP.

When the wind speed is 120 km/h, the deformation of TWMP is shown in Fig 13a. The deformation of region S2 is the largest, and the deformation in region S1 and region S2 is symmetrically distributed along the Y-axis. The maximum deformation in region S2 is 7.7 mm, and that in region S1 is 5.6 mm. The deformation contour of TWMP is similar to the noise contour in Fig 6b.

**Fig 14:**
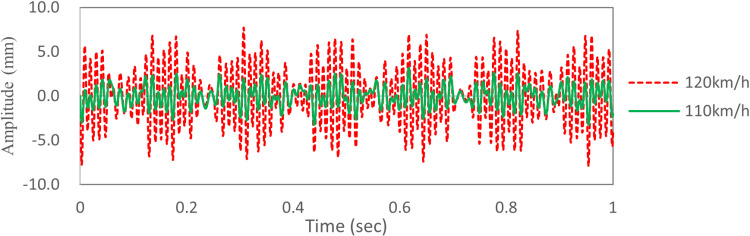
The time domain data of deformation in region S2.

The time domain data of deformation in region S2 is shown in Fig 14. The deformation in region S2 at 120 km/h is significantly larger than that at 110km/h. The deformation curve at 120 km/h shows a “beat” shape, with a maximum value of 7.7 mm. The deformation curve at 110 km/h is flat, and the maximum value is 3.1 mm. Since there is no booming noise under the condition of 110 km/h wind speed, the maximum deformation of TWMP of 3.0 mm is selected as the optimization objective.

## Noise control

The deformation analysis results show that the deformation of TWMP is much larger than its thickness under the action of high-speed airflow, so the small deformation theory is no longer applicable. Therefore, the large deformation theory is used to analyze the vibration of TWMP. In this part, the dynamic simulation model of TWMP is established, and the dynamic characteristics are analyzed.

### Dynamic characteristic analysis of TWMP

**Fig 15:**
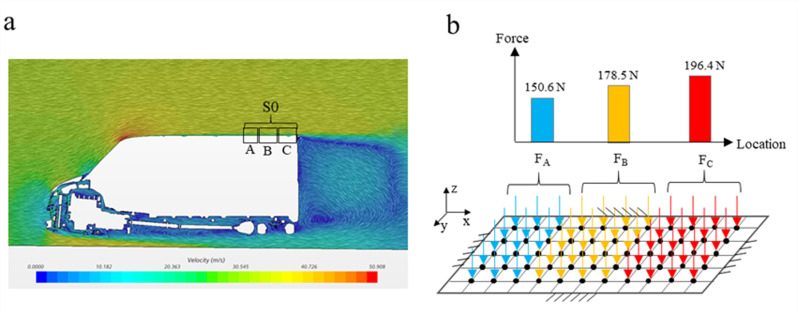
CFD simulation results. (a) The flow field (b) The normal force in region S0.

The input of the dynamic simulation is the normal force of the TWMP. In order to calculate the normal force in region S0 under the action of high-speed airflow, the CFD model of the body is established, and the flow field simulation is carried out at 120 km/h [22]. The simulation results are shown in Fig 15. Fig 15a shows the flow field distribution on the body surface. There is obvious airflow disturbance at the rear end of the body, which causes the normal force fluctuation and aggravates TWMP vibration in region S0. The region S0 is divided into three parts along the X direction, namely, part A, part B and part C. The load on the three parts is shown in Fig 15b. The loads on part A, part B and part C are 150.6 N, 178.5 N and 196.4 N respectively.

**Fig 16:**
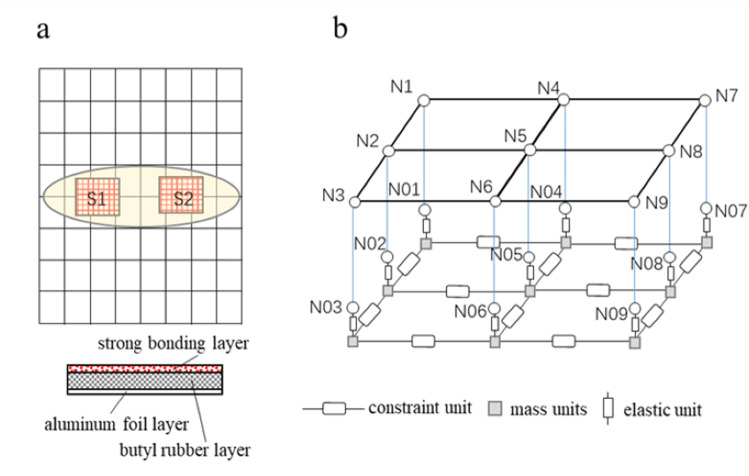
Damping plate. (a) Attachment position (b) Equivalent model of damping plate.

According to the deformation results in Fig 13, the attachment positions of the damping plate are region S1 and region S2, as shown in Fig 16a. The damping plate is composed of aluminum foil layer, butyl rubber layer and strong bonding layer. The butyl rubber layer provides damping and the strong bonding layer provides restraint. The equivalent model of the damping plate is shown in Fig 16b, which is composed of multiple mass units, elastic units and constraint units. The sheet metal attached to the damping plate is equally divided into four parts, and the endpoints of each part are represented by N1 - N9. The auxiliary nodes N01 - N09 are created by translating each endpoint distance d1 along the normal direction, and d1 is the thickness of the damping plate.

A rigid connection is created between Ni and N0i (i = 1,2,..., 9) to ensure that Ni and N0i have the same motion state. The mass unit m_0i_ (i = 1,2,...,9) is created below the auxiliary node. An elastic element is created between N0i and M0i with a stiffness of k_0i_ and a damping of C_0i_. The tensile stiffness of the damping plate is K_L_ and the damping is C. A constraint element is created between adjacent mass units, whose stiffness is the shear stiffness K_G_ of the damping plate. There is


$$\{\begin{array}{l} m_{0i}=\frac{1}{i}M\\ k_{0i}=\frac{1}{i}K_{L}\\ c_{0i}=\frac{1}{i}C \end{array}(i=1,2,\cdots \cdots ,9)$$ (6)

Since the surface of TWMP is attached with damping plate, the effect of damping on vibration should be considered. Therefore, damping force needs to be added to Eq. (4), there is


$$\frac{Eh^{3}}{12(1-\mu^{2})}(\frac{\partial^{2}}{\partial x^{2}}+\frac{\partial^{2}}{\partial y^{2}})\omega +C\frac{\partial \omega }{\partial t}+m\frac{\partial^{2}\omega }{\partial t^{2}}=q(x,y,t)$$ (7)

In Fig 16b, the mass unit and the elastic unit constitute a dynamic vibration absorber, which can absorb the vibration energy of TWMP, and the dynamic equation is


$$m_{0i}\overset{..}{z}(t)+C_{0i}\dot{z}(t)+k_{0i}z(t)=q(t)$$ (8)

where, z(t) stands for the displacement of mass unit.

Kinetic energy E_I_, elastic potential energy E_S_ and damping energy energy E_D_ are calculated by integrating z(t) in Eq. (8), there is


$$\begin{array}{c} E=E_{I}+E_{D}+E_{S}=\int \left[m_{0i}\overset{..}{z}(t)+c_{0i}\dot{z}(t)+kz(t)\right]dz(t)\\ =\int m_{0i}\overset{..}{z}(t)\dot{z}(t)dt+\int c_{0i}\dot{z}(t)\dot{z}(t)dt+\int k_{0i}z(t)\dot{z}(t)dt\\ =\frac{1}{2}m_{0i}\dot{z}{\left(t\right)}^{2}+\int c_{0i}\dot{z}{\left(t\right)}^{2}dt+\frac{1}{2}k_{0i}z{\left(t\right)}^{2}\\ =\int q(t)dz(t) \end{array}$$ (9)

E_I_ and E_S_ are alternating between increasing and decreasing in the time domain. E_D_ increases with time and plays the role of energy dissipation. The elastic modulus E of the rubber layer, the area A_0_ and the thickness d_0_ affect the stiffness K_L_ and damping C of the damping plate, there is


$$\{\begin{array}{l} K_{L}=\frac{EA_{0}}{d_{0}}=n\frac{EA^{'}}{d_{0}}=nK_{L}^{'}\\ C=C^{'}\frac{A_{0}}{A^{'}}=nC^{'}\\ K_{G}=nK_{G}^{'} \end{array}$$ (10)

where, A’ stands for the area of the square damping plate specimen (22500 mm^2^), C’ stands for the damping coefficient of the specimen, K_L_’ stands for the tensile stiffness of the specimen, K_G_’ stands for the shear stiffness of the specimen, and n stands for the amplification coefficient.

Eq. (10) shows that the tensile stiffness of the damping plate is proportional to E and A_0_, and inversely proportional to d_0_. The damping of the damping plate is proportional to E and d_0_. The damping effect is related to the area of damping pale, the larger the area, the better the damping effect. However, the amount of damping plate should not be too much, otherwise it is not conducive to the lightweight design of the body. The structural parameters of the damping plate specimen used in the paper are shown in Table 2.

**Table 2:** Parameters of damping plate specimen.

Parameter	K’_L_ (N/mm)	K’_G_ (N/mm)	d_0_ (mm)	M’ (kg)	C’
Value	10.8	22.4	2	0.25	0.165

**Fig 17:**
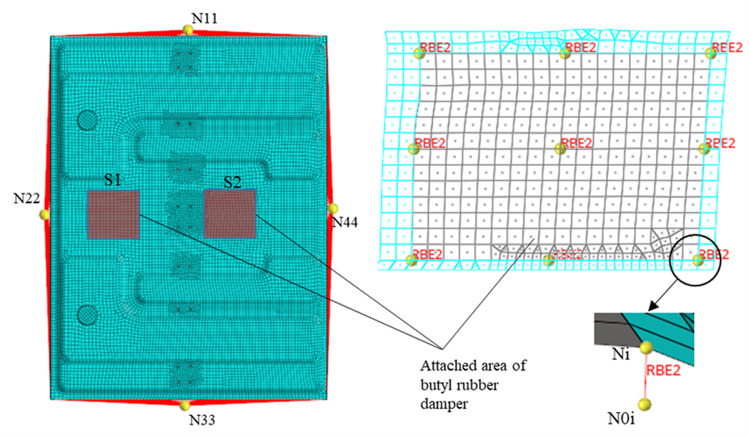
Finite element model of TWMP.

The finite element model of TWMP is established in Hyperworks [23]. Constraint nodes N11, N22, N33, and N44 are created below the center of the four sides of the TWMP. The rigid connection between the constrained node and each side nodes is established respectively. The auxiliary nodes in regions S1 and S2 are created according to Fig 16b. A rigid connection between Ni and N0i is created, as shown in Fig 17. The damping plate is 150mm in length and 150mm in width. The modal neutral file (MNF) of the TWMP is calculated in Hyperworks.

**Fig 18:**
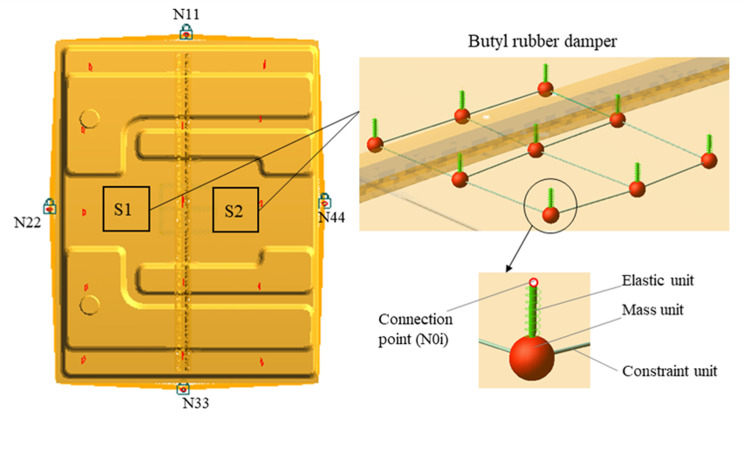
Dynamic model of TWMP.

The MNF file is imported into ADAMS to create the dynamic model of the TWMP [24], as shown in Fig 18. Fixed hinges are created on nodes N11, N22, N33, and N44, respectively. The damping plate equivalent model is created according to the method in Fig 16b, and the parameters are assigned to each unit according to Eq. (6) and Eq. (10).

The exciting force is applied to the TWMP according to the method in Fig 15b, and the expression is


$$\{\begin{array}{l} F_{A}=8.4sin\left(304.236t\right)+9.6sin\left(403.77t\right)+11.7sin\left(548.376t\right)+9.5sin\left(587.814t\right)\\ F_{B}=9.3sin\left(304.236t\right)+8.2sin\left(403.77t\right)+13.5sin\left(548.376t\right)+11.2sin\left(587.814t\right)\\ F_{C}=10.2sin\left(304.236t\right)+8.7sin\left(403.77t\right)+14.2sin\left(548.376t\right)+11.6sin\left(587.814t\right) \end{array}$$ (11)

where, t stands for time.

**Fig 19:**
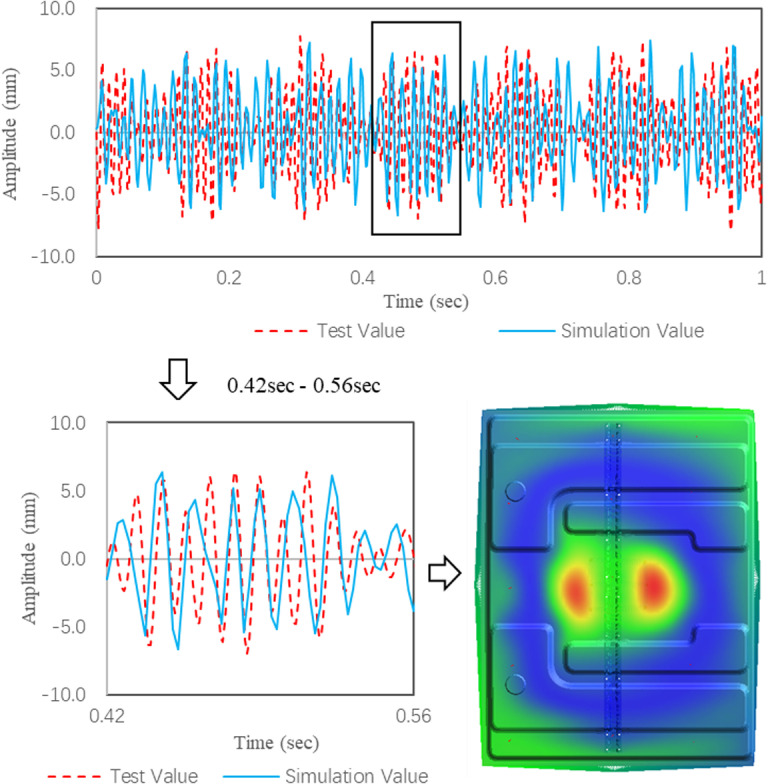
Vibration comparison data in the time domain.

The parameters of the mass unit, elastic unit and constraint unit are set to “0”, that is, the dynamic simulation is carried out under the condition of no damping plate. The simulation results are compared with the experimental results to verify the accuracy of the TWMP dynamic model. The comparison results in time domain are shown in Fig 19. The two curves are in good agreement, the simulation maximum value is 6.7 mm and the test maximum value is 7.7 mm, and the error is 8.7%. The TWMP vibration simulation contour is consistent with the test results in Fig 13.

**Fig 20:**
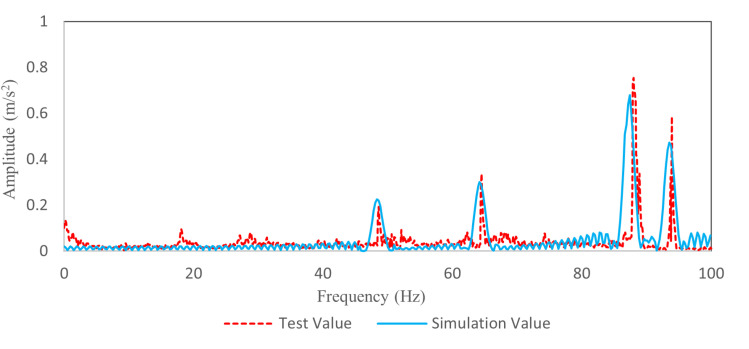
Vibration comparison data in the frequency domain.

Fig 20 shows the comparison results in the frequency domain. The peak frequencies of the simulation are 48.5 Hz, 64.7 Hz, 87.7 Hz and 93.7 Hz. The simulation results are consistent with the test results. The comparison results show that the TWMP dynamic model is correct.

### Area design of damping plate

The three elements of optimization include design variable, objective function and constraint equation [25]. The mass, tensile stiffness, shear stiffness and damping coefficient in the damping model are defined as design variables, which are calculated according to Eq. (6) and Eq. (10). Therefore, the design variables are optimized by changing the amplification factor n. The maximum deformation of TWMP at 110 km/h is selected as the objective function, that is, f(max) = 3 mm. The use of damping plates not only to achieve the effect of vibration reduction, but also to consider the lightweight of the body. Therefore, the damping plate area is selected as the constraint condition, that is, 0 ≤ S ≤ 2S0 (0 ≤ n ≤ 2).

**Fig 21:**
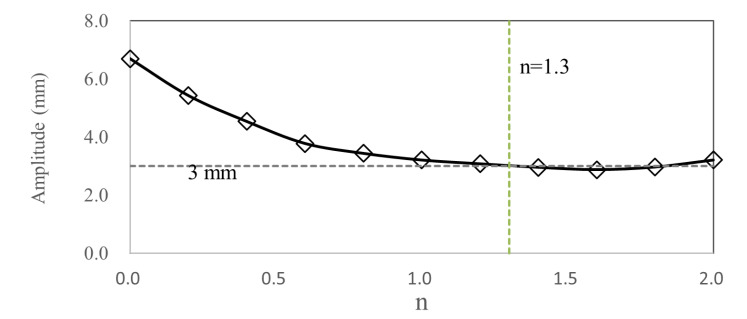
The maximum deformation of TWMP with different n.

Fig 21 shows the relationship curve between the maximum deformation of TWMP and n, which presents nonlinear characteristics. When n = 1.3, the maximum deformation is 3.0 mm, and the corresponding damping plate area is 38025 mm^2^. When n >  1.5, the damping effect is basically unchanged. Therefore, the area of square damping plate is selected as 40000 mm^2^.

**Fig 22:**
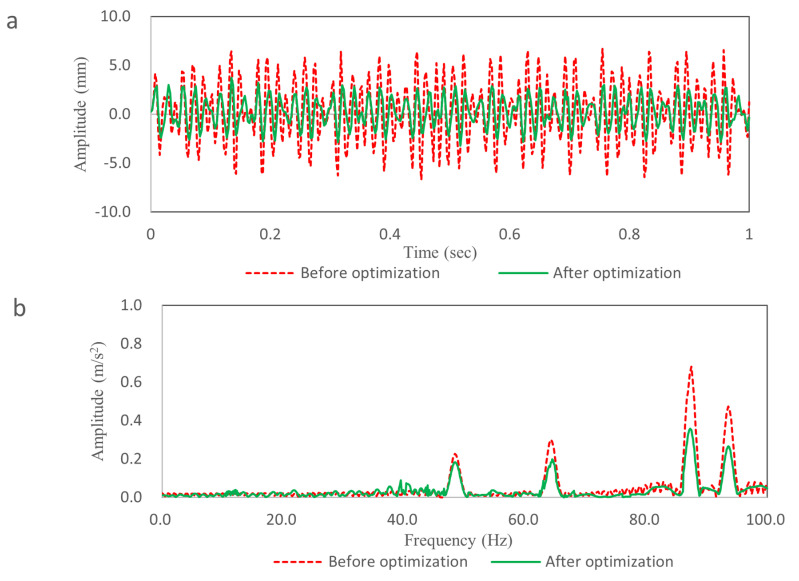
Simulation comparison results. (a) The time domain curve. (b) The frequency domain curve.

Fig 22a shows the simulation comparison results in time domain. The maximum deformation before optimization is 6.7 mm, the maximum deformation after optimization is 2.95 mm, the vibration attenuation rate is 55.5%, and the fluctuation is significantly reduced. Fig 22b shows the simulation comparison results in frequency domain. Compared with the result before optimization, the peak decreases of 48.5 Hz, 64.7 Hz, 87.7 Hz and 93.7 Hz are 11.1%, 25.0%, 47.1% and 43.5%, respectively.

The simulation results show that the choice of the attached position and attached area can significantly reduce the vibration of TWMP, especially the damping effect of high frequency vibration is more obvious.

## Validation test

**Fig 23:**
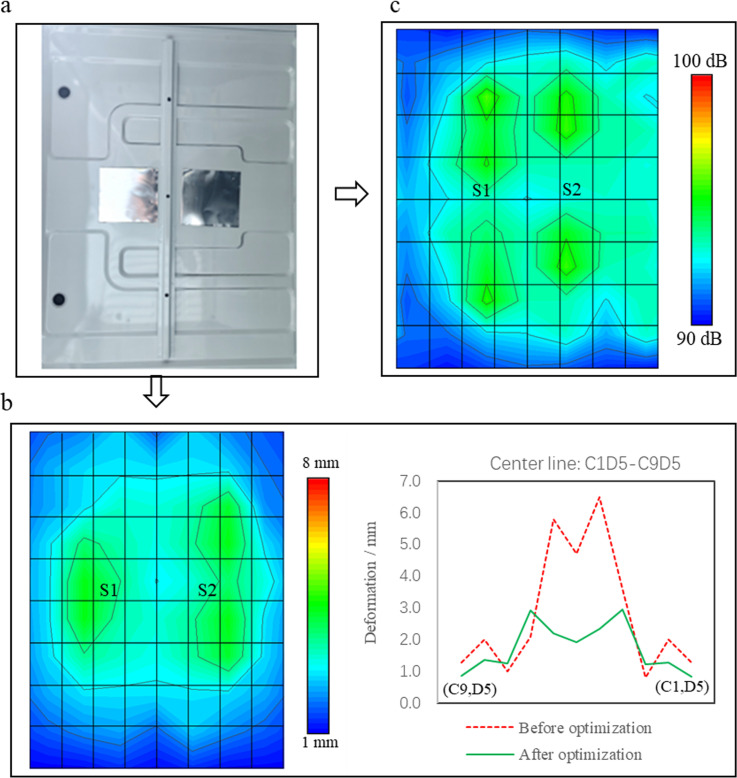
Test comparison results. (a) The test photograph. (b) The deformed data. (c) The sound field data.

According to the simulation optimization scheme, the damping plates are attached to regions S1 and S2, as shown in Fig 23a. Fig 23b shows the deformation of TWMP at 120 km/h. Compared with the result in Fig 12, the deformation decreases significantly, and the maximum deformation is still distributed near regions S1 and S2. The maximum deformation in region S1 is 2.93 mm, which decreases by 49.5%. The maximum deformation in region S2 is 2.96 mm, which decreases by 54.5%.

Fig 23c shows the sound field contour of TWMP at 120 km/h. Compared with the result in Fig 5b, the noise is significantly reduced, the maximum noise is 93.3 dB, and the noise is reduced by 6.5 dB. The maximum noise is no longer in the regions S1 and S2, and shifts to both sides of the center line C1D5-C9D5.

**Fig 24:**
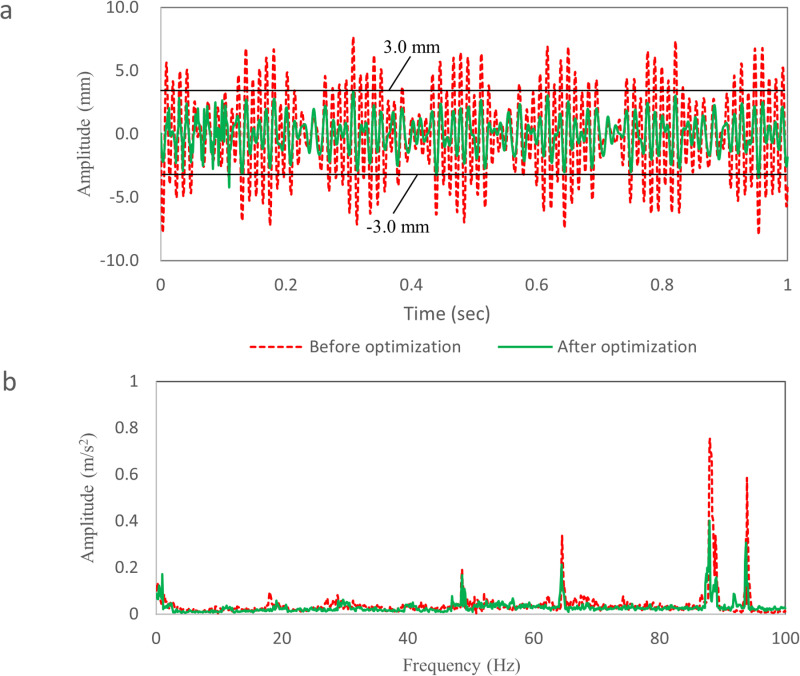
Comparison results of vibration test data. (a) The time domain data. (b) The frequency domain data.

Fig 24a shows the time-domain vibration curve of TWMP at 120 km/h. The vibration data after optimization is a random signal, and there is no “beat” phenomenon on the curve, and the amplitude is in the range of -3.0 mm—3.0 mm. Compared with the results before optimization, the average damping rate is more than 50.0%.

Fig 24b shows the frequency domain vibration curve of TWMP at 120 km/h. The damping plate does not change the natural frequency of the TWMP. Compared with the data before optimization, the peak value corresponding to 48.5 Hz, 64.7 Hz, 87.7 Hz and 93.7 Hz decreases by 13.0%, 27.9%, 45.8% and 47.5%, respectively. The simulation results are consistent with the test results.

**Fig 25:**
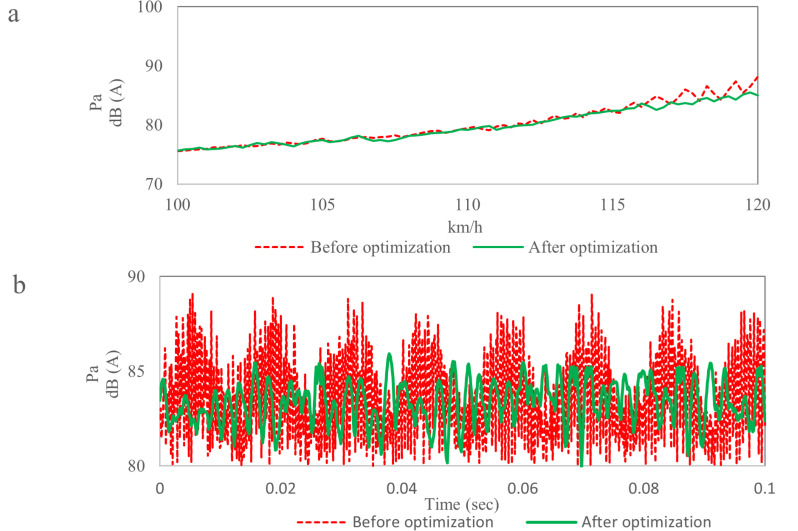
Noise comparison results near the driver’s ear. (a) Noise data under uniform acceleration conditions. (b) Noise data at 120 m/h.

Fig 25 shows the noise comparison results near the driver’s ear. Fig 25a shows the noise under uniform acceleration condition. When the wind speed is less than 115 km/h, The optimization scheme has little effect on noise. When the wind speed is higher than 115 km/h, the noise data after optimization is significantly lower than that before optimization, the average reduction of noise is 3.0 dB, and the fluctuation of noise data is small after optimization.

Fig 25b shows the noise near the driver’s ear at 120 km/h. There is no “beat” phenomenon on the curve after optimization, the noise data is presented as a random signal, and there is no booming noise near the driver’s ear. The maximum noise before and after optimization is 89.2 dB and 85.8 dB respectively, and the noise reduction is 3.4 dB.

**Fig 26:**
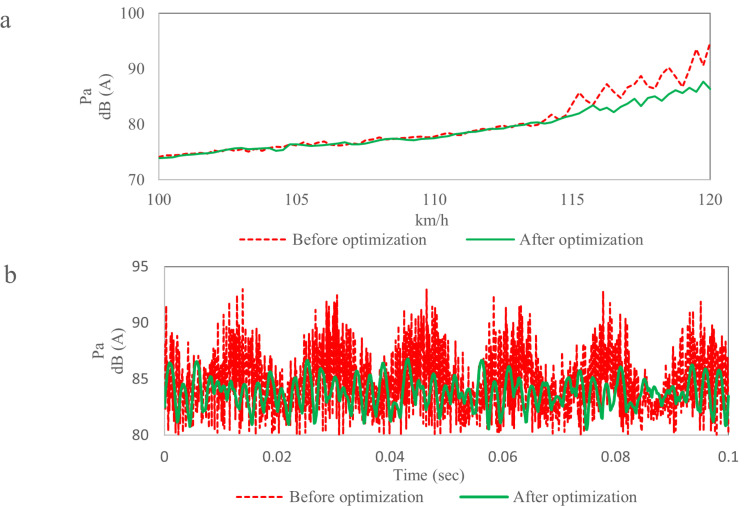
Noise comparison results near the passenger’s ear. (a) Noise data under uniform acceleration conditions. (b) Noise data at 120 kmh.

Fig 26 shows the noise comparison results near the passenger’s ear. The noise curve under uniform acceleration condition as shown in Fig 26a. When the wind speed is less than 115 km/h, the change of noise is not obvious. When the wind speed is higher than 115 km/h, the noise data after optimization is significantly lower than that before optimization, the average reduction is 4.0 dB, and the fluctuation of noise data after optimization is small.

Fig 26b shows the noise data near passenger’s ear at 120 km/h. The “beat” phenomenon disappears after optimization, the noise curve is relatively stable, and there is no booming noise. The maximum noise before optimization is 92.9 dB, the maximum noise after optimization is 86.6 dB, and the average reduction is 6.3 dB.

The test results show that the method in the paper can effectively identify and solve the problem of booming noise in vehicle under the action of high-speed airflow. The simulation results are consistent with the test results, which shows that the modeling method of damping plate is correct, and the attachment position and attachment area of damping plate can be analyzed quickly and accurately by using the method.

## Conclusion

The high-speed airflow directly acts on the roof of the body, causing the TWMP to vibrate, which produces booming noise in vehicle. The identification of problem frequency and noise source is studied through the vibration and noise test. Through the simulation of the TWMP structure mode and the acoustic cavity mode, the cause of the booming noise is analyzed. The comparison of noise at different wind speeds shows that the booming noise is not only related to the sound-structure coupling, but also to the vibration intensity of the TWMP. In this paper, a damping plate is used to reduce the vibration amplitude of the TWMP to solve the booming noise.

A dynamic model of damping plate is established, which consists of mass unit, elastic unit and constraint unit. The simulation results are in good agreement with the experimental results, which shows that the dynamic modeling is correct. The damping coefficient, attachment position and attachment area of the damping plate can be analyzed quickly and accurately by using the method.

Based on experimental, finite element, dynamic and fluid techniques, a method for identification, analysis and optimization of TWMP vibration and noise under high-speed airflow is presented in this paper. This method can effectively identify and solve the problem of the booming noise, and has important engineering application value.

## Supporting information

S1 Data Noise data. (XLSX)
